# Effect of Extraction Solvents on Total Polyphenolic Content and Antioxidant Capacity of *Syzygium Aromaticum* L. Flower Bud from Ethiopia

**DOI:** 10.1155/2022/4568944

**Published:** 2022-11-23

**Authors:** Shibiru Temesgen, J. M. Sasikumar, Meseret C. Egigu

**Affiliations:** ^1^Department of Biology, Oda Bultum University, Ethiopia; ^2^School of Biological Sciences and Biotechnology, College of Natural and Computational Sciences, Haramaya University, Haramaya, P.O. Box 138, Ethiopia

## Abstract

In this study, the antioxidative activity and polyphenolic content of *Syzygium aromaticum*'s flower bud were compared under different extraction solvents including chloroform, ethyl acetate, methanol, and aqueous. The antioxidant activity was assessed *via* established *in vitro* assay models such as 2, 2-diphenyl-1-Picrylhydrazyl (DPPH) radical scavenging assay, NO^−^ radical scavenging assay, H_2_O_2_ scavenging assay and Fe^3+^ reducing capacity. Total phenolic content was measured according to Folin–Ciocalteu's method, and total flavonoid content was estimated by using the aluminum chloride colorimetric method. The results showed that aqueous extract possessed the highest TPC (19.11 ± 2.76 mg GAE/g DW) and TFC (15.32 ± 1.53 mg CtE/g DW). Among the extracts, methanol extract exerted the strongest radical DPPH quenching activity with an IC_50_ value of 303.56 ± 13.14 *μ*g/mL. The highest NO^−^ radical scavenging activity was shown by methanol extract (IC_50_192.94 ± 1.9 *μ*g/mL) which is stronger than BHT (IC_50_247.64 ± 12.89 *μ*g/mL). Methanol extract showed a strong H_2_O_2_ scavenging activity (IC_50_233.71 ± 3.72 *μ*g/mL). The highest Fe^3+^ reducing capacity was shown by methanol extract (Absorbance = 0.36 ± 0.05). Strong and positive correlations were observed between total phenolic and flavonoid contents and the antioxidant assays. The results of the present work revealed that the tested spice demonstrated high antioxidant activity, total phenolics, and flavonoids. Thus, this spice is worth considering as important source of natural antioxidant agents.

## 1. Introduction

Key threats to biomolecules of the cells are free radicals in the form of reactive oxygen species (ROS) and reactive nitrogen species (RNS). They are the root cause for several degenerative ailments in humans [1]. Antioxidants are known to hamper cells' damage mediated by the oxidizing free radical chain reactions [2]. One of the sources of antioxidants are spices that are used to flavor foods since antiquity. Epidemiological studies validated the role of spice consumption to prevent the degenerative diseases like cardiovascular diseases, carcinogenesis, and atherosclerosis [3]. Spices have been considered as one among topmost five foods with the highest polyphenol compounds such as flavonoids, phenolic acids, and tannins [4–7]. Many previous studies [8–13] have shown antioxidant properties of spices.

The secondary metabolites, especially phenolics, have antioxidant property [14, 15]. Phenolic compounds (including flavonoids) of edible plants are the most abundant secondary metabolites in the plant kingdom, they play a protective role as nonenzymatic antioxidants in cells, protecting cells from oxidative stress damage, and are rich sources of human health-promoting metabolites [16, 17]. The type of solvent used to extract secondary metabolites has impact on their quality and quantity [18, 19]. The influence of extracting solvents on total phenolics and antioxidant activities have also been reported by different authors [20–22]. The chemical components of the extracts and essential oils are dependent upon the species, genotype, environment, organ type, developmental phase, extraction methods, and other variables [17].

Ethiopia is an abode of a variety of spices and condiments like *Cinnamomum zeylanicum*, *Lippia adoensis*, *Myristica fragrans*, *Nigella sativa*, *Piper capense*, *Rhamnus prinoides*, *Ruta chalepensis*, *Syzygium aromaticum*, *Thymus shimperi*, and *Trachyspermum ammi*. These spices have been used to improve taste and aroma of several local dishes [23, 24]. The tested spice in the present study, *Syzygium aromaticum* (Myrtaceae) locally known as *kerunfud*, is one of the spices used in the preparation of Ethiopian local dishes like “Mekelesha” and “Nitirkibie” [24] and “wot” [23]. In addition to its aroma and taste enhancing property, it is also used to treat tooth ache, wounds, warts, and erectile dysfunction [25, 26] in Ethiopia. The buds of *S. Aromaticum* are rich in eugenol, eugenyl acetate and *β*-caryophyllene [27]. Phenolic acids such as hydroxibenzoic acids, hydroxicinamic acids, and hydroxiphenyl propens, caffeic, ferulic, elagic, and salicylic acids were also isolated from extracts of *S. aromaticum* [28]. Buds of *S. aromaticum* exhibited antihyperglycemic, hypolipidemic, and hepatoprotective activities [29]. Previously, *S. aromaticum* hydrosols and essential oil were investigated for antimicrobial activity [30, 31] in Ethiopia. Across the world, studies have been conducted on antioxidant activity of *S. aromaticum* [32–38]. Though most of the above mentioned Ethiopian spices were studied for their antioxidant activity [13], to the best of our knowledge, Ethiopian cultivars of *S. aromaticum* have not yet been evaluated to relate extraction solvent to the quantity of phenolic compounds and antioxidant capacity. Thus, we hypothesized that different solvents yield in different amounts of phenolics to exhibit varying antioxidant activities. Our objective of this study was to quantify total phenolic and flavonoid contents of the flower bud of *S. aromaticum* extracted by using different solvents and evaluate their antioxidant activities.

## 2. Materials and Methods

### 2.1. Chemicals

Chloroform, ethyl acetate, methanol, ferric chloride (FeCl_3_), aluminum chloride (AlCl_3_), sodium hydroxide (NaOH), sodium carbonate (Na_2_CO_3_), sodium nitroprusside, hydrogen peroxide (H_2_O_2_), butylated hydroxytoluene (BHT), trichloroacetic acid (TCA), potassium ferricyanide [K_3_Fe_3_(CN)_6_], and N-1-naphthyl ethylene diamine dihydrochloride, catechin, and gallic acid, and 2,2-diphenyl-2-picrylhydrazyl (DPPH) were procured from Merck Co. India. All chemicals are of analytical grade.

### 2.2. Spice Extract Preparation

Flower buds were collected from Bate village around Haramaya University (9°23' N of latitude and 42° 01' E of longitude), Eastern Hararghe, Ethiopia. The collected material was identified by experts in the School of Biological Sciences and Biotechnology and its voucher specimen was kept in the herbarium of Haramaya University. The collected flower buds were air-dried in a hot air oven (Bluefic, India) at 40°C for 96 hours and then pulverized using mortar and pestle. The powder of the spice (50°g) was extracted (in triplicate) by separately dissolving in 250 ml of chloroform, ethyl acetate, methanol, and distilled water for 24 hours. The extract was filtered using Whatman no. 1 filter paper and the filtered solvent was evaporated under reduced pressure using a rotary evaporator (Heidolph rotary evaporator, Laborata 4001) at 55°C to remove the solvent. The obtained crude extracts were stored at -4°C until further analysis. The obtained extracts were labeled as chloroform extract (CE), ethyl acetate extract (EAE), methanol extract (ME), and aqueous extract (AQE).

### 2.3. Quantitative Analysis of Total Polyphenolic Compounds

#### 2.3.1. Total Phenolic Quantification

The total phenolic content in the extracts was determined according to the Folin–Ciocalteu procedure [39]. About 4 mg of the dried crude extracts were mixed with 5 ml 80% acetone, shaken well in a vortex-shaker, and centrifuged at 2,200 × *g* for 2 min at room temperature. The supernatant was transferred to a 10 mL volumetric flask with a Pasteur pipette. The remaining residue was extracted twice with 2.5 mL 80% acetone, shaken well in a vortex-shaker, and centrifuged as before after standing for 5 min, and the supernatants were transferred to the same 10 mL volumetric flask with a Pasteur pipette. Aliquots (100 *μ*L) of supernatant from each sample were put into a 10 mL volumetric flask and mixed with 1.9 mL deionized water. The Folin–Ciocalteu–phenol reagent (1 mL) was added and the solution was shaken vigorously and mixed with 5 mL sodium carbonate (20%). After 20 min, the solution was centrifuged at 2,200 × *g* for 2 min at room temperature. Absorbance at 735 nm was measured in a spectrophotometer (Shimadzu UV-2401PC) and the results were expressed as gallic acid equivalents from a gallic acid standard curve (mg GAE/g Extract DW; *r*^2^ = 0.9867). The analyses were performed in triplicate.

#### 2.3.2. Total Flavonoid Quantification

The total flavonoid content in the extracts was determined by aluminum chloride colorimetric method based on the method indicated by Ordonez et al. [40]. Briefly, a volume of 0.5 mL of AlCl_3_ ethanol solution (2%) was added to 0.5 mL of extract. After one hour incubation at room temperature, the absorbance was measured at 420 nm using UV-Vis spectrophotometer. The analysis was performed in triplicate. The total flavonoid content was estimated from a catechin standard curve and the results are expressed as mg catechin equivalents (mg CtE/g Extract DW; r^2^ = 0.9675).

### 2.4. Assessment of Antioxidant Capacity

#### 2.4.1. Scavenging Capacity on DPPH Stable Radical

The determination of DPPH stable radical scavenging activities of the extracts and standard were evaluated based on the method described by Singh et al. [41]. Extracts (1 mL) of the different concentrations (i.e., 25, 50, 100, 200, 400, 800, and 1000 *μ*g/mL^−1^) made by reconstituting in respective solvents were added to DPPH solution (5 mL, 0.1 mM) in methanol and vortexed. After 20 minutes of reaction at 25°C, the absorbance was measured at 517 nm against a blank (methanol) in a UV-Vis spectrophotometer (Shimadzu UV-2401PC). Methanolic DPPH solution (5 ml) without antioxidant was used as control. The DPPH scavenging activity of the extract was expressed as IC_50_ (inhibitory concentration), that is, the concentration of the extract at which DPPH radicals were scavenged by 50%. Butyl hydroxy toluene (BHT) was used as standard antioxidant.

The percentage quenching of DPPH was calculated as follows:

(1) $$\begin{array}{c} \text{DPPH}∙\text{quenching capacity }\left(\%\right)=\frac{\left(\text{Abs control}\right)-\left(\text{Abs sample}\right)}{\text{Abs control}}\text{ X }100. \end{array}$$

where Abs sample is the absorbance of the sample (extract and standard antioxidant) and Abs control is DPPH solution without the added extract.

#### 2.4.2. Scavenging Capacity on Nitric Oxide Radical

Nitric oxide (NO) generated from sodium nitroprusside (SNP) in aqueous solution at physiological pH was estimated by the use of the Griess reaction [42]. The reaction mixture (3 mL) containing SNP (10 mM, 2 mL), phosphate buffer saline (0.5 mL, pH 7.4), and the extracts (0.5 mL) at different concentrations (25, 50, 100, 200, 400, 800, and 1000 *μ*g mL^−1^) were incubated at 25°C for 150 min. After incubation, 0.5 mL of the incubated solution containing nitrite was pipetted and mixed with 1 mL of sulfanilic acid reagent (0.33% in 20% glacial acetic acid) and allowed to stand for 5 min for completing diazotization. Then, 1 mL of N-(1-naphthyl) ethylenediamine dihydrochloride was added, mixed, and allowed to stand at 25°C for 30 min. The absorbance of pink colored chromophore formed during diazotization was measured at 540 nm. The NO scavenging activity of the extract was expressed as IC_50_ (inhibitory concentration), that is, the concentration of the extract at which NO radicals were quenched by 50%. Butyl hydroxy toluene (BHT) was used for comparison.

(2) $$\begin{array}{c} \text{Nitric oxide scavenging capacity }\left(\%\right)=\frac{\left(\text{Abs control}\right)-\left(\text{Abs sample}\right)}{\text{Abs control}}\text{ X }100 \end{array}$$

where Abs sample is absorbance of the sample and Abs control is absorbance of control.

#### 2.4.3. Scavenging Capacity on H_2_O_2_

Scavenging capacity of the extracts towards Hydrogen peroxide (H_2_O_2_) was carried out by adopting the method of Ruch et al. [43]. Solution of H_2_O_2_ (50 mM) was prepared in phosphate buffer (50 mM, pH 7.4). Extracts' concentrations (25, 50, 100, 200, 400, 800, and 1000 *μ*g mL^−1^, 0.3 mL) were mixed separately with H_2_O_2_ solution (0.6 ml). The resulting solution was kept for 10 minutes and its absorbance was measured at 230 nm thereafter. The H_2_O_2_ scavenging activity of the extract was expressed as IC_50_ (inhibitory concentration), that is, the concentration of the extract at which H_2_O_2_ radicals were quenched by 50%. Butyl hydroxy toluene (BHT) was used for comparison.

(3) $$\begin{array}{c} {\text{H}}_{2}{\text{O}}_{2}\text{ scavenging capacity }\left(\%\right)=\frac{\left(\text{Abs control}\right)-\left(\text{Abs sample}\right)}{\text{Abs control}}\text{ X }100 \end{array}$$

where Abs_sample_ is absorbance of the sample and Abs_control_ is absorbance of control.

#### 2.4.4. Reducing Capacity of Fe^3+^

Reducing capacity of the spice's extracts towards Fe^3+^ was performed according to Oyaizu [44]. From extracts' solution (25, 50, 100, 200, 400, 800, and 1000 *μ*g mL^−1^), 0.5 mL portion was mixed with 2.5 mL of sodium phosphate buffer (0.2 M, pH 6.6) and 2.5 mL of potassium ferricyanide [K_3_Fe_3_(CN)_6_] (1%) in test tubes. The resulting solution was vortexed and incubated at 50°C for 20 min. Then, 2.5 mL of trichloroacetic acid (TCA) (10%, w/v) was added to all tubes and the solutions were centrifuged at 3000 × *g* for 10 min. The aqueous solution of FeCl_3_ (1%, 1 ml) was diluted by adding deionized water (5 mL). To this solution, the upper layer of the centrifuged solution (5 mL) was mixed and incubated at 35°C for 10 min. Absorbance of the developed color was measured at 700 nm. BHT was used as standard. Reducing capacity of the spice extracts towards Fe^3+^ was interpreted from relation of absorbance and reducing capacity: when absorbance increases, reducing capacity also increases.

### 2.5. Statistical Analysis

Triplicates were made for the experiments. Statistical Package for Social Sciences version 20 and Microsoft excel were used for data analysis. One-way analysis of variance (ANOVA) with Duncan's multiple range tests was used for data analysis. A statistically significant difference was considered at *p* < 0.05. The Pearson correlation coefficient (squared > 0.700) was used to analyze correlation between the amounts of TPC, TFC, and antioxidant capacities.

## 3. Results

### 3.1. Total Phenolic and Total Flavonoid Contents

Results showed that TPC and TFC from *S. aromaticum* extracts significantly (*p* < 0.05) varied between extraction solvents used. AQE had the highest TPC and TFC values followed by ME, EAE, and CE (Table 1).

### 3.2. Antioxidant Capacity

#### 3.2.1. Scavenging Capacity on DPPH Stable Radical

**Figure 1:**
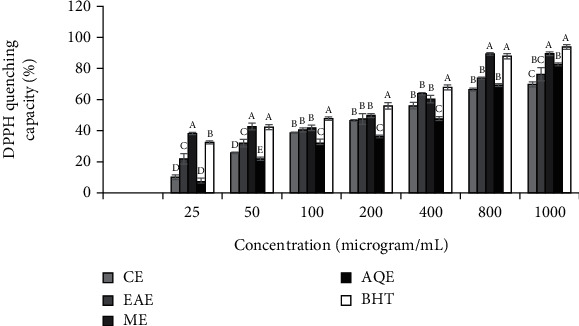
DPPH radical scavenging capacity of extracts of *S. aromaticum* flower buds. Values are Mean ± SE, *n* = 3.

The DPPH radical scavenging capacity of each extract type (i.e., CE, EAE, ME, and AQE) from *S. aromaticum* significantly (*p* < 0.05) varied between the different concentrations. DPPH scavenging capacity also varied significantly (*p* < 0.05) between the extraction solvents used to get the extracts (Figure 1). The DPPH scavenging activity of CE (*r*^2^ = 0.885, *p* = 0.008), EAE (*r*^2^ = 0.931, *p* = 0.002), ME (*r*^2^ = 0.988, *p* ≤ 0.001), and AQE (*r*^2^ = 0.970, *p* ≤ 0.001) showed strong positive correlations with total phenolic contents. Likewise, the DPPH scavenging activity of CE (*r*^2^ = 0.992, *p* = 0.078), EAE (*r*^2^ = 0.927, *p* = 0.073), ME (*r*^2^ = 0.746, *p* = 0.254), and AQE (*r*^2^ = 0.896, *p* = 0.104) had also strong positive correlations with total flavonoid contents, though values were not statistically significant. Assessment by IC_50_ values showed significant (*p* < 0.05) variation between extracts in their DPPH scavenging activity with values in a decreasing order of scavenging activity being ME (IC_50_ = 303.56 ± 13.14 *μ*g/mL) > EAE (IC_50_ = 413.14 ± 10.52 *μ*g/mL) > AQE (IC_50_ = 502.40 ± 10.17 *μ*g/mL) > CE (IC_50_ = 509.48 ± 9.88 *μ*g/mL). The scavenging activity of all extracts, however, was found to be lower than the standard antioxidant, BHT, with an IC_50_ value of 256.38 ± 25.41 *μ*g/mL.

#### 3.2.2. Scavenging Capacity on NO^−^ Radical

**Figure 2:**
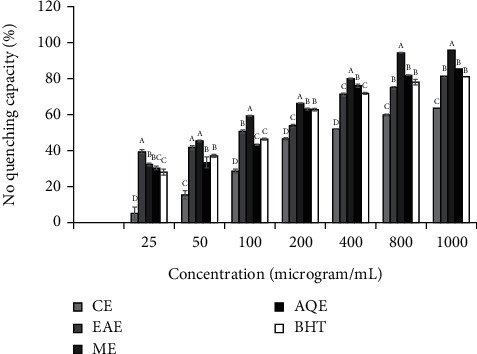
NO^−^ scavenging capacity of extracts of *S. aromaticum* flower buds. Values are Mean ± SE, *n* = 3.

The NO^−^ radical inhibition potentials of each extract type (i.e., CE, EAE, ME, and AQE) varied significantly (*p* < 0.05) between the different concentrations, and inhibitory effects of extracts increased with concentration (Figure 2). Extraction solvent used to get the extracts had also significantly impacted (*p* < 0.05) NO^−^ radical inhibition potential. Strong positive correlations were observed between the NO^−^ radical scavenging activity of the extracts: CE: *r*^2^ = 0.884, *p* = 0.012; EAE: *r*^2^ = 0.940, *p* = 0.002; ME: *r*^2^ = 0.916, *p* = 0.004; AQE: *r*^2^ = 0.895, *p* = 0.006; total phenolic contents. The total flavonoid contents also showed strong correlations with NO^−^ radical scavenging activity of CE (*r*^2^ = 0.942, *p* = 0.058), EAE (*r*^2^ = 0.992, *p* = 0.008), ME (*r*^2^ = 0.941, *p* = 0.059), and AQE (*r*^2^ = 0.914, *p* = 0.086). Assessment by IC_50_ values showed significant (*p* < 0.05) variation between extracts in their NO^−^ radical inhibition activity with values in a decreasing order of NO^−^inhibition activity being ME (IC_50_ = 192.94 ± 1.9 *μ*g/mL) > EAE (IC_50_ = 290.37 ± 3.20 *μ*g/mL) > AQE (IC_50_ = 296.16 ± 6.72 *μ*g/mL) > CE (IC_50_ = 605.84 ± 5.36 *μ*g/mL). The NO^−^ scavenging activity of extracts, except ME, was found to be lower than the standard antioxidant, BHT, with an IC_50_ value of 247.64 ± 12.89 *μ*g/mL.

#### 3.2.3. H_2_O_2_ Scavenging Capacity

**Figure 3:**
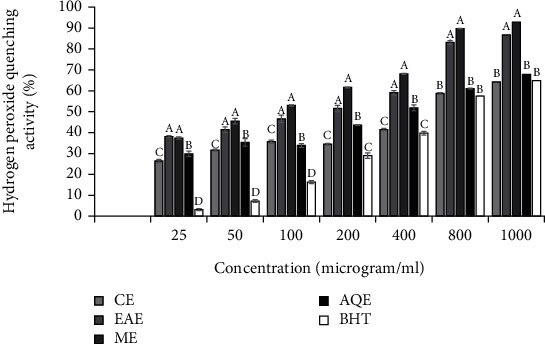
H_2_O_2_ scavenging capacity of extracts of *S. Aromaticum* flower buds. Values are Mean ± SE, *n* = 3.

With regard to H_2_O_2_ scavenging activity of the extracts of *S. aromaticum* flower bud, significant difference was observed between the concentrations of each extract type and scavenging activity increased with extract concentrations (Figure 3). Extraction solvent used to get the extracts had also significantly influenced (*p* < 0.05) H_2_O_2_ scavenging capacity. The H_2_O_2_ scavenging capacity of CE (*r*^2^ = 0.988, *p* ≤ 0.001), EAE (*r*^2^ = 0.992, *p* ≤ 0.001), ME (*r*^2^ = 0.969, *p* ≤ 0.001), and AQE (*r*^2^ = 0.977, *p* ≤ 0.001) exhibited strong positive correlation with total phenolic contents. Strong positive correlations were also found between the H_2_O_2_ scavenging capacity of CE (*r*^2^ = 0.823, *p* = 0.177), EAE (*r*^2^ = 0.960, *p* = 0.040), ME (*r*^2^ = 0.934, *p* = 0.066), and AQE (*r*^2^ = 0.728, *p* = 0.272) and total flavonoid contents. Assessment by IC_50_ values exhibited significant (*p* < 0.05) variation between extracts in their H_2_O_2_ scavenging capacity with values in a decreasing order of scavenging activity being ME (IC_50_ = 233.71 ± 3.72 *μ*g/mL) > EAE (IC_50_ = 312.98 ± 23.15 *μ*g/mL) > AQE (IC_50_ = 532.61 ± 25.16 *μ*g/mL) > CE (IC_50_ = 621.84 ± 9.65 *μ*g/mL). The H_2_O_2_ scavenging capacity of BHT (IC_50_ = 683.42 ± 11.27 *μ*g/mL) was found to be lower than the extracts.

#### 3.2.4. Reducing Capacity of Fe^3+^

**Figure 4:**
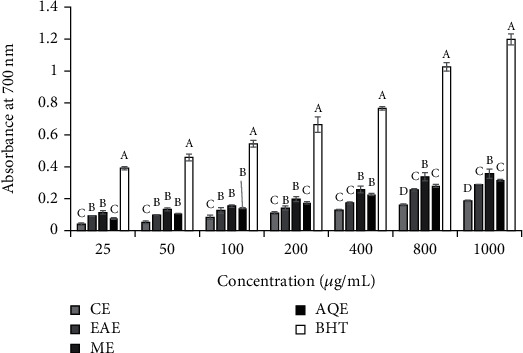
Reducing capacity of extracts of *S. aromaticum* flower buds. Values are Mean ± SE, *n* = 3.

There was significant (*p* < 0.05) difference between the concentration of each extract type and between the different solvents used to get the extracts in reducing ferric ion (Fe^3+^) to ferrous ion (Fe^2+^) (Figure 4). Among the extracts, ME of *S. aromaticum* exhibited the highest reducing power, whereas CE was the least. Strong positive correlations were observed between total phenolic contents of CE (*r*^2^ = 0.0.953, *p* ≤ 0.001), EAE (*r*^2^ = 0.0.994, *p* ≤ 0.001), ME (*r*^2^ = 0.0.978, *p* ≤ 0.001), and AQE (*r*^2^ = 0.0.970, *p* ≤ 0.001) and reducing capacity. The Fe^3+^ reducing capacity by the extracts in a decreasing order was ME >AQE > EAE > CE based on the absorbance value. High absorbance value indicates high reducing capacity. The reducing capacity of the standard antioxidant BHT (absorbance 1.20 ± 0.06) was found to be higher than the extracts.

## 4. Discussion

### 4.1. Total Phenolic and Flavonoid Contents

Despite an ample number of studies on this spice across the world, *S. aromaticum* from Ethiopia was examined for the first time for its antioxidant capacity and polyphenolic content. The secondary metabolite profile, which in turn influences antioxidant activity, could differ between regions due to growth environmental factors and plants' genotypes [17]. In this study, the total phenolic and flavonoid values significantly varied between extraction solvents. The highest values were recorded in AQE followed by ME, EAE, and CE. The extraction solvents used were of varying polarity, with water being the highest polar solvent and chloroform the least polar solvent. Therefore, the variation observed between the different solvents extracts is attributed to their polarity difference. Previously, Egigu et al. [45] reported that the extraction potential of solvents depend on their polarity strength. Several studies have proved the significance of high polar organic solvents for the improvement of extraction yield of phenolic compounds [46–48]. Highly hydroxylated aglycone forms of phenolic compounds are soluble in high polar solvents such as water and alcohols (ethanol and methanol) and hydroxy groups of phenolic compounds contribute to antioxidant activity [49]. Concomitant with variations in values of total phenolics and total flavonoids, the antioxidant capacity of the tested plant also varied with the solvents used to extract. Correlation analysis also showed strong positive correlation between total phenolic and flavonoid contents of the extracts and the different antioxidant assays, suggesting that differences of antioxidant capacities between the extracts can be ascribed to polyphenolic contents. Previously, several researchers [50, 51] showed the presence of strong correlation between antioxidant capacities and the plant's total phenolic contents that confirms phenolic compounds as important contributors to antioxidant activities. These plant-derived versatile products are in high demand for cosmetics, food, and pharmaceutical industries due to their safety, being less prone to risk, relatively low extraction cost, easy storage of products, and ease of scaling-up [17].

### 4.2. Antioxidant Capacity

In this study, four different antioxidant assays, namely, DPPH (2, 2-diphenyl-2-picrylhydrazine) radical scavenging, NO^−^ radical inhibition, H_2_O_2_ scavenging capacity, and Fe^3+^ reducing capacity were conducted to evaluate the antioxidant potential of the extracts of the *S. aromaticum* flower bud. This is so the antioxidant property of the compounds in a crude extract may vary depending on the nature of oxidants [52]. Our results showed that all solvents' extracts of *S. aromaticum* flower bud significantly scavenged DPPH radical in a concentration dependent manner. The control solution without the extracts, however, had no inhibitory effect at all. Previously, Molyneux [53] and Adefegha and Oboh [54] explained that the DPPH radical scavenging assay is a commonly used cost-effect approach of evaluating the antioxidant property of secondary metabolites in crude plant extracts. The antioxidant property of natural products, for example, phenolic compounds is ascribed to electron donation in the form of hydrogen to DPPH radical [55]. Pietta [56] also reported that the antioxidant property of phenolic compounds due to their redox properties that enable them act as reducing agents, hydrogen donors, and/or singlet oxygen quenchers.

Comparison between the different solvents' extracts showed that ME had the highest DPPH radical scavenging capacity, which is on a par with that of the standard BHT followed by EAE. The DPPH radical quenching capacities of AQE and CE, however, were less than that of ME and CE. The same was also verified by their IC_50_ values. Here, it is noteworthy that extracts obtained by highly polar solvent (water) and highly nonpolar solvent (chloroform) had lesser power of responding to DPPH radical. This may be related to the variation in secondary compounds profile of the different solvents' extracts that should be verified through GC-MS and HPLC analyses. According to Egigu et al. [45], solvents with different polarity show difference in their extraction potential, which in turn determine the quantity and quality of compounds extracted. Previous studies [9, 35] on *S. aromaticum* also confirmed that extracts of highly polar solvents such as ethanol and water possess strong DPPH scavenging activity. Generally, the DPPH radical scavenging capacities of extracts showed significant positive correlation with the amounts of total phenolics and flavonoids measured. Earlier, Adebiyiet al. [57], Sasikumar et al. [13], and Soulef et al. [58] reported a strong positive correlation between *in vitro* antioxidant activity and phenolic contents of plant extracts.

Compared to the control (solution without extracts) all solvents' extracts of *S. aromaticum* significantly scavenged nitric oxide (NO^−^) in a concentration dependent manner. Nitric oxide (NO^−^) is physiologically important molecule that reacts with different molecules such as proteins and DNA [59]. Coupled with superoxide anion radical (O_2_^∗^^−^), nitric oxide forms peroxynitrite anion (ONOO−), which is more potent Reactive Nitrogen Species (RNS) resulting in nitrosative stress [60]. Plant natural products, for example, phenolics compete for superoxide with nitric oxide and prevent nitrosative stress caused by peroxynitrite anion [61]. In the present study, the *in vitro* nitric oxide scavenging capacity varied with the extract type where ME had the highest value followed by EAE, AQE, and CE. Our results of ME, which was on a par with the standard, BHT, accords with that of Sasikumar et al. [13]. We also noticed strong positive correlation between nitric oxide scavenging capacity and total phenolics of the extracts. Our finding is also supported by that of Sasikumar et al. [13] who evaluated the nitric oxide scavenging capacities of different spices.

Though a weak oxidant, H_2_O_2_ can oxidize and inactivate some enzymes as it is. However, it may react with some cations such as Fe^2+^ and Cu^2+^ within a cell and produce a toxic hydroxyl radical (OH•) that oxidizes many cellular molecules [62]. Results of this study showed that all solvents' extracts of *S. aromaticum* scavenged H_2_O_2_ and the scavenging capacity increased with extracts' concentration. Highest scavenging capacity was by ME followed by EAE, AQE, and CE. Hydrogen peroxide scavenging capacities of ME, EAE, and AQE were superior to the standard used at all concentration levels while that of CE was higher than the standard at concentrations below 100 *μ*g/mL and on a par at concentrations ≥200 *μ*g/mL. Formerly, a study by [54] vindicated the effect of high polar solvent extracts of *S. aromaticum* for Fe^3+^ reduction. Moreover, a study by [63] showed the effective scavenging activity of clove (*S. aromaticum*) oil against H_2_O_2_. Scavenging capacities of extracts correlated positively with total phenolic content, suggesting phenolic compounds are reductants responsible to scavenge H_2_O_2_ [56]. *In vitro* reducing power of a plant extract can be considered as an indicator for the presence of potential antioxidant compounds. In this study, the reducing power of extracts was determined by the intensity of the resultant Prussian blue color complex which absorbs at 700 nm. Our result suggests that all solvents' extracts have antioxidant compounds that convert Fe^3+^ to Fe^2+^ and reducing power of extracts appeared to increase with extracts' concentration. However, the extracts had significantly lower reducing power when compared to the standard.

## 5. Conclusions

In conclusion, different extraction solvents resulted in different concentrations of TPC and TFC with the highest values measured in strongly polar solvents. The antioxidant properties of the extracts showed strong positive correlation with TPC and TFC, suggesting that the difference in antioxidant properties of extracts of *S. aromaticum* flower bud is attributed to polyphenolic content.

## Figures and Tables

**Table 1:** Total phenolic and flavonoid contents of different solvents' extracts of *S. aromaticum* flower buds.

Extract solvent	TPC (mg GAE/g)	TFC (mg CtE/g)
Chloroform extract (CE)	6.23 ± 1.09c	5.63 ± 1.08c
Ethyl acetate extract (EAE)	7.98 ± 1.25bc	5.83 ± 1.17c
Methanol extract (ME)	10.74 ± 2.00b	13.25 ± 1.95b
Aqueous extract (AQE)	19.11 ± 2.76a	15.32 ± 1.53a

Note: the values are mean ± standard deviation (*n* = 3). Small letter superscripts compare means in a column. Means with similar letters are not varied significantly, whereas means with different letters are significantly varied at *p* < 0.05. GAE: gallic acid equivalents; CtE: catechin equivalents.

## Data Availability

All relevant data are included in the paper.
